# (*Z*)-3-(3,4-Dimeth­oxy­phen­yl)-3-(4-fluoro­phen­yl)-1-morpholino­prop-2-en-1-one

**DOI:** 10.1107/S1600536811022008

**Published:** 2011-06-22

**Authors:** Baoshan Chai, Changling Liu

**Affiliations:** a State Key Laboratory of the Discovery and Development of Novel Pesticides, Shenyang Research Institute of Chemical Industry, Shenyang 110021, People’s Republic of China

## Abstract

The title compound, C_21_H_22_FNO_4_, is an isomer of flumorph (systematic name 4-[3-(3,4-dimethoxyphenyl)-3-(4-fluorophenyl)-1-oxo-2-propenyl]morpholine), which was developed by Shenyang research institute of chemical industry and used as fungicide. The mol­ecule adopts a *Z* configuration about the C=C double bond. The dihedral angle between the two benzene rings is 73.45 (11)°.

## Related literature

The title compound is an isomer of flumorph. For background to the use of flumorph as a fungicide, see: Liu *et al.* (2002 ▶). For the synthesis, see: Li *et al.* (2000 ▶). For the use of flumorph, see: Liu (2000 ▶).
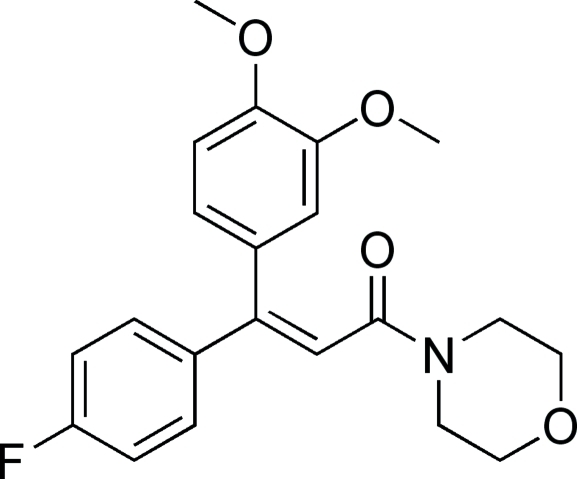


## Experimental

### 

#### Crystal data

C_21_H_22_FNO_4_
*M* *_r_* = 371.40
Monoclinic, 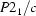

*a* = 6.4963 (18) Å
*b* = 13.306 (4) Å
*c* = 20.890 (6) Å
β = 95.651 (4)°
*V* = 1797.0 (9) Å^3^
*Z* = 4
Mo *K*α radiation
μ = 0.10 mm^−1^
*T* = 113 K
0.20 × 0.18 × 0.10 mm

#### Data collection

Rigaku Saturn CCD area-detector diffractometer
Absorption correction: multi-scan (*CrystalClear*; Rigaku, 2002) ▶ *T*_min_ = 0.980, *T*_max_ = 0.990
15064 measured reflections
3171 independent reflections
2713 reflections with *I* > 2σ(*I*)
*R*_int_ = 0.066

#### Refinement

*R*[*F* ^2^ > 2σ(*F* ^2^)] = 0.062
*wR*(*F* ^2^) = 0.115
*S* = 1.19
3171 reflections
246 parameters
H-atom parameters constrained
Δρ_max_ = 0.18 e Å^−3^
Δρ_min_ = −0.23 e Å^−3^

### 

Data collection: *CrystalClear* (Rigaku, 2002 ▶); cell refinement: *CrystalClear*; data reduction: *CrystalClear*; program(s) used to solve structure: *SHELXS97* (Sheldrick, 2008 ▶); program(s) used to refine structure: *SHELXL97* (Sheldrick, 2008 ▶); molecular graphics: *SHELXTL* (Sheldrick, 2008 ▶); software used to prepare material for publication: *CrystalStructure* (Rigaku, 2002 ▶).

## Supplementary Material

Crystal structure: contains datablock(s) I. DOI: 10.1107/S1600536811022008/zk2011sup1.cif

Structure factors: contains datablock(s) I. DOI: 10.1107/S1600536811022008/zk2011Isup2.hkl

Supplementary material file. DOI: 10.1107/S1600536811022008/zk2011Isup3.cml

Additional supplementary materials:  crystallographic information; 3D view; checkCIF report

## Figures and Tables

**Table 1:** Hydrogen-bond geometry (Å, °)

*D*—H⋯*A*	*D*—H	H⋯*A*	*D*⋯*A*	*D*—H⋯*A*
C19—H19*A*⋯O2^i^	0.99	2.57	3.279 (3)	129
C11—H11⋯O2^ii^	0.95	2.58	3.478 (3)	158
C15—H15⋯O3^iii^	0.95	2.38	3.216 (3)	147
C21—H21*A*⋯O3^iv^	0.99	2.42	3.105 (3)	125
C8—H8*B*⋯O4^v^	0.98	2.55	2.983 (3)	106

Symmetry codes: (i) 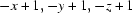; (ii) 
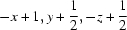
; (iii) 
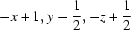
; (iv) 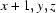; (v) 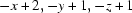.

**Fig. 1.**
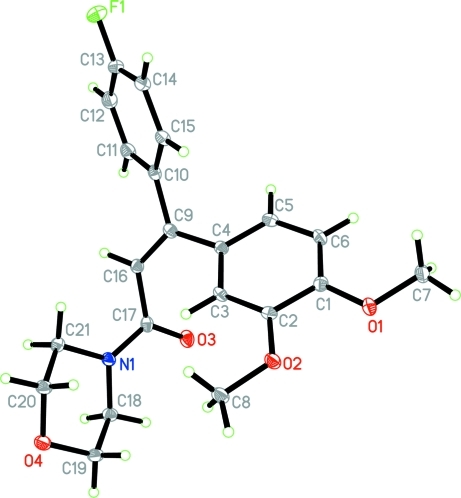
The molecular structure of (I), with atom labels and 30% probability displacement ellipsoids.

## supplementary crystallographic information

### Comment

Flumorph is a commercial fungicide, it was discovered in 1994. As reported (Li *et al.*, 2000), there are two isomers in the product. In this paper, we report the crystal structure of one of the isomer. In the crystal structure of the title compound, the molecule adopts a *Z* configuration about the C=C double bond and The dihedral angle between the two benzene rings is 73.45 (11)°.

### Experimental

The title compound was synthesized by (3,4-dimethoxyphenyl)(4-fluorophenyl)methanone with 1-morpholinoethanone under basic condition in toluene. The crude products were purified by recrystallization from methanol and then grown from DMF to afford colorless single crystals suitable for X-ray diffraction. To a solution of sodium *tert*-butoxide(0.15 mol) in toluene(280 ml) and *tert*-butanol(30 ml) was added the (3,4-Dmethoxyphenyl)(4-fluorophenyl)methanone (26 g, 0.1 mol) at room tempreture. The reaction mixture was heated to reflux for 6 h, a solution of 1-morpholinoethanone(0.15 mol) in toluene(20 ml) was added over 1 h. The *tert*-butanol was distilled off at the same time, then the mixture was washed with water, the organic extracts were dried over magnesium sulfate and concentrated. Afford the tittle product compound as a white solid(31.5 g, 88%).

Anal. Calcd for C_21_H_22_FNO_4_: C, 67.91; H, 5.97; N, 3.77. Found: C, 67.93; H, 5.94; N, 3.75. ^1^H NMR(CDCl_3_): 3.19 (m, 2H, morpholine-CH_2_), 3.29 (m, 2H, morpholine-CH_2_), 3.52 (m, 4H, morpholine-2CH_2_), 3.82 (s, 3H, OCH_3_), 3.90 (s, 3H, OCH_3_), 6.24 (s, 1H, CH), 6.77 (s, 1H, Ph—H), 6.81 (s, 1H, Ph—H), 6.82 (s, 1H, Ph—H), 7.07 (m, 2H, Ph-2H), 7.28 (m, 2H, Ph-2H).

### Refinement

Although all H atoms were visible in difference maps, they were finally placed in geometrically calculated positions, with C—H distances in the range 0.95–0.99 Å, and included in the final refinement in the riding model approximation,with *U*_iso_(H) = 1.3*U*_eq_(C) and *U*_iso_(H) = 1.7*U*_eq_(C).

### Crystal data

**Table:**

C_21_H_22_FNO_4_	*F*(000) = 784
*M**_r_* = 371.40	*D*_x_ = 1.373 Mg m^−^^3^
Monoclinic, *P*2_1_/*c*	Mo *K*α radiation, λ = 0.71073 Å
Hall symbol: -P 2ybc	Cell parameters from 5162 reflections
*a* = 6.4963 (18) Å	θ = 1.8–27.9°
*b* = 13.306 (4) Å	µ = 0.10 mm^−^^1^
*c* = 20.890 (6) Å	*T* = 113 K
β = 95.651 (4)°	Prism, colorless
*V* = 1797.0 (9) Å^3^	0.20 × 0.18 × 0.10 mm
*Z* = 4	

### Data collection

**Table:**

Rigaku Saturn CCD area-detector diffractometer	3171 independent reflections
Radiation source: rotating anode	2713 reflections with *I* > 2σ(*I*)
multilayer	*R*_int_ = 0.066
Detector resolution: 14.63 pixels mm^-1^	θ_max_ = 25.0°, θ_min_ = 1.8°
ω and φ scans	*h* = −7→7
Absorption correction: multi-scan (*CrystalClear*; Rigaku, 2002)	*k* = −15→15
*T*_min_ = 0.980, *T*_max_ = 0.990	*l* = −24→24
15064 measured reflections	

### Refinement

**Table:**

Refinement on *F*^2^	Primary atom site location: structure-invariant direct methods
Least-squares matrix: full	Secondary atom site location: difference Fourier map
*R*[*F*^2^ > 2σ(*F*^2^)] = 0.062	Hydrogen site location: inferred from neighbouring sites
*wR*(*F*^2^) = 0.115	H-atom parameters constrained
*S* = 1.19	*w* = 1/[σ^2^(*F*_o_^2^) + (0.0358*P*)^2^ + 0.3441*P*] where *P* = (*F*_o_^2^ + 2*F*_c_^2^)/3
3171 reflections	(Δ/σ)_max_ = 0.004
246 parameters	Δρ_max_ = 0.18 e Å^−^^3^
0 restraints	Δρ_min_ = −0.23 e Å^−^^3^

### Special details

**Table:**

Geometry. All e.s.d.'s (except the e.s.d. in the dihedral angle between two l.s. planes) are estimated using the full covariance matrix. The cell e.s.d.'s are taken into account individually in the estimation of e.s.d.'s in distances, angles and torsion angles; correlations between e.s.d.'s in cell parameters are only used when they are defined by crystal symmetry. An approximate (isotropic) treatment of cell e.s.d.'s is used for estimating e.s.d.'s involving l.s. planes.
Refinement. Refinement of *F*^2^ against ALL reflections. The weighted *R*-factor *wR* and goodness of fit *S* are based on *F*^2^, conventional *R*-factors *R* are based on *F*, with *F* set to zero for negative *F*^2^. The threshold expression of *F*^2^ > σ(*F*^2^) is used only for calculating *R*-factors(gt) *etc*. and is not relevant to the choice of reflections for refinement. *R*-factors based on *F*^2^ are statistically about twice as large as those based on *F*, and *R*- factors based on ALL data will be even larger.

### Fractional atomic coordinates and isotropic or equivalent isotropic displacement parameters (Å^2^)

**Table:**

	*x*	*y*	*z*	*U*_iso_*/*U*_eq_	
F1	0.8136 (2)	0.40799 (13)	−0.02485 (8)	0.0508 (5)	
O1	0.0240 (2)	0.26719 (12)	0.34490 (8)	0.0267 (4)	
O2	0.3744 (2)	0.32110 (12)	0.40261 (8)	0.0248 (4)	
O3	0.4465 (2)	0.66020 (12)	0.32079 (8)	0.0283 (4)	
O4	1.0853 (2)	0.61491 (12)	0.46137 (8)	0.0264 (4)	
N1	0.7861 (3)	0.63993 (14)	0.35473 (9)	0.0206 (5)	
C1	0.1421 (3)	0.32636 (17)	0.30920 (12)	0.0214 (5)	
C2	0.3331 (3)	0.35649 (17)	0.34125 (11)	0.0194 (5)	
C3	0.4633 (3)	0.41694 (16)	0.31010 (11)	0.0196 (5)	
H3	0.5914	0.4374	0.3321	0.024*	
C4	0.4115 (3)	0.44909 (17)	0.24662 (11)	0.0196 (5)	
C5	0.2246 (3)	0.41741 (17)	0.21506 (11)	0.0219 (6)	
H5	0.1879	0.4370	0.1717	0.026*	
C6	0.0898 (3)	0.35698 (17)	0.24639 (11)	0.0224 (6)	
H6	−0.0386	0.3368	0.2244	0.027*	
C7	−0.1556 (3)	0.21977 (18)	0.31262 (12)	0.0282 (6)	
H7A	−0.2509	0.2713	0.2938	0.042*	
H7B	−0.2247	0.1797	0.3436	0.042*	
H7C	−0.1139	0.1759	0.2785	0.042*	
C8	0.5605 (3)	0.35492 (19)	0.43833 (12)	0.0263 (6)	
H8A	0.6800	0.3322	0.4170	0.039*	
H8B	0.5687	0.3273	0.4820	0.039*	
H8C	0.5603	0.4285	0.4404	0.039*	
C9	0.5592 (3)	0.51373 (17)	0.21513 (11)	0.0204 (5)	
C10	0.6104 (3)	0.48695 (17)	0.14952 (12)	0.0216 (5)	
C11	0.6589 (4)	0.56046 (19)	0.10521 (12)	0.0284 (6)	
H11	0.6452	0.6295	0.1155	0.034*	
C12	0.7261 (4)	0.5340 (2)	0.04685 (13)	0.0343 (7)	
H12	0.7575	0.5842	0.0170	0.041*	
C13	0.7468 (4)	0.4343 (2)	0.03280 (12)	0.0335 (7)	
C14	0.6999 (4)	0.3593 (2)	0.07389 (12)	0.0296 (6)	
H14	0.7154	0.2907	0.0628	0.036*	
C15	0.6292 (3)	0.38614 (18)	0.13209 (12)	0.0237 (6)	
H15	0.5929	0.3350	0.1606	0.028*	
C16	0.6596 (3)	0.59017 (17)	0.24583 (11)	0.0206 (5)	
H16	0.7663	0.6210	0.2248	0.025*	
C17	0.6207 (3)	0.63187 (17)	0.31001 (12)	0.0206 (5)	
C18	0.7732 (4)	0.69660 (18)	0.41400 (11)	0.0240 (6)	
H18A	0.6262	0.7082	0.4205	0.029*	
H18B	0.8403	0.7629	0.4104	0.029*	
C19	0.8775 (3)	0.64070 (19)	0.47103 (11)	0.0253 (6)	
H19A	0.8772	0.6831	0.5100	0.030*	
H19B	0.7987	0.5787	0.4782	0.030*	
C20	1.0895 (4)	0.55327 (19)	0.40550 (11)	0.0248 (6)	
H20A	1.0116	0.4906	0.4114	0.030*	
H20B	1.2344	0.5348	0.4001	0.030*	
C21	0.9958 (3)	0.60684 (18)	0.34551 (12)	0.0236 (6)	
H21A	1.0820	0.6656	0.3366	0.028*	
H21B	0.9918	0.5608	0.3082	0.028*	

### Atomic displacement parameters (Å^2^)

**Table:**

	*U*^11^	*U*^22^	*U*^33^	*U*^12^	*U*^13^	*U*^23^
F1	0.0442 (10)	0.0796 (13)	0.0310 (10)	−0.0077 (9)	0.0152 (8)	−0.0142 (9)
O1	0.0226 (9)	0.0285 (10)	0.0285 (10)	−0.0089 (8)	0.0010 (8)	−0.0016 (8)
O2	0.0257 (9)	0.0275 (10)	0.0201 (10)	−0.0066 (8)	−0.0029 (7)	0.0026 (7)
O3	0.0192 (9)	0.0276 (10)	0.0380 (11)	0.0026 (8)	0.0021 (8)	−0.0069 (8)
O4	0.0256 (9)	0.0316 (10)	0.0213 (10)	0.0032 (8)	−0.0014 (7)	−0.0037 (8)
N1	0.0176 (10)	0.0233 (11)	0.0206 (11)	0.0028 (9)	−0.0003 (9)	−0.0033 (9)
C1	0.0205 (13)	0.0187 (12)	0.0256 (14)	0.0001 (10)	0.0049 (11)	−0.0027 (10)
C2	0.0230 (13)	0.0177 (12)	0.0170 (13)	0.0029 (10)	−0.0009 (10)	−0.0014 (10)
C3	0.0188 (12)	0.0163 (12)	0.0227 (14)	−0.0014 (10)	−0.0034 (10)	−0.0037 (10)
C4	0.0202 (13)	0.0180 (12)	0.0202 (13)	0.0003 (10)	0.0000 (10)	−0.0029 (10)
C5	0.0261 (14)	0.0204 (13)	0.0185 (13)	0.0011 (11)	−0.0018 (11)	−0.0009 (10)
C6	0.0184 (12)	0.0228 (13)	0.0249 (14)	−0.0011 (11)	−0.0040 (11)	−0.0037 (11)
C7	0.0211 (13)	0.0285 (15)	0.0354 (16)	−0.0043 (11)	0.0050 (12)	−0.0068 (12)
C8	0.0268 (14)	0.0300 (14)	0.0208 (14)	−0.0030 (12)	−0.0042 (11)	0.0018 (11)
C9	0.0186 (12)	0.0199 (13)	0.0217 (14)	0.0028 (10)	−0.0029 (10)	0.0010 (10)
C10	0.0174 (12)	0.0236 (13)	0.0228 (14)	−0.0013 (10)	−0.0035 (10)	0.0020 (11)
C11	0.0266 (14)	0.0302 (15)	0.0272 (15)	−0.0040 (12)	−0.0028 (12)	0.0035 (12)
C12	0.0297 (15)	0.0480 (18)	0.0247 (15)	−0.0094 (14)	0.0005 (12)	0.0066 (13)
C13	0.0254 (15)	0.0548 (19)	0.0206 (15)	−0.0055 (13)	0.0039 (11)	−0.0094 (13)
C14	0.0252 (14)	0.0347 (15)	0.0285 (15)	−0.0015 (12)	0.0008 (12)	−0.0095 (12)
C15	0.0231 (14)	0.0243 (14)	0.0229 (14)	−0.0033 (11)	−0.0022 (11)	−0.0015 (11)
C16	0.0175 (12)	0.0233 (13)	0.0205 (13)	0.0010 (11)	0.0000 (10)	0.0023 (11)
C17	0.0187 (13)	0.0148 (12)	0.0283 (15)	−0.0019 (10)	0.0020 (11)	0.0002 (10)
C18	0.0254 (14)	0.0253 (14)	0.0210 (14)	0.0034 (11)	0.0015 (11)	−0.0039 (11)
C19	0.0264 (14)	0.0306 (14)	0.0190 (14)	0.0023 (12)	0.0030 (11)	−0.0033 (11)
C20	0.0239 (13)	0.0283 (14)	0.0218 (14)	0.0044 (11)	0.0007 (11)	−0.0046 (11)
C21	0.0194 (13)	0.0289 (14)	0.0228 (14)	0.0032 (11)	0.0029 (11)	−0.0025 (11)

### Geometric parameters (Å, °)

**Table:**

F1—C13	1.365 (3)	C8—H8C	0.9800
O1—C1	1.370 (3)	C9—C16	1.338 (3)
O1—C7	1.435 (3)	C9—C10	1.485 (3)
O2—C2	1.367 (3)	C10—C15	1.398 (3)
O2—C8	1.430 (3)	C10—C11	1.404 (3)
O3—C17	1.235 (3)	C11—C12	1.380 (3)
O4—C19	1.427 (3)	C11—H11	0.9500
O4—C20	1.429 (3)	C12—C13	1.368 (4)
N1—C17	1.357 (3)	C12—H12	0.9500
N1—C18	1.459 (3)	C13—C14	1.370 (4)
N1—C21	1.462 (3)	C14—C15	1.388 (3)
C1—C6	1.384 (3)	C14—H14	0.9500
C1—C2	1.409 (3)	C15—H15	0.9500
C2—C3	1.376 (3)	C16—C17	1.495 (3)
C3—C4	1.402 (3)	C16—H16	0.9500
C3—H3	0.9500	C18—C19	1.508 (3)
C4—C5	1.389 (3)	C18—H18A	0.9900
C4—C9	1.489 (3)	C18—H18B	0.9900
C5—C6	1.398 (3)	C19—H19A	0.9900
C5—H5	0.9500	C19—H19B	0.9900
C6—H6	0.9500	C20—C21	1.516 (3)
C7—H7A	0.9800	C20—H20A	0.9900
C7—H7B	0.9800	C20—H20B	0.9900
C7—H7C	0.9800	C21—H21A	0.9900
C8—H8A	0.9800	C21—H21B	0.9900
C8—H8B	0.9800		
			
C1—O1—C7	118.03 (19)	C10—C11—H11	119.5
C2—O2—C8	117.29 (17)	C13—C12—C11	118.9 (2)
C19—O4—C20	110.37 (17)	C13—C12—H12	120.6
C17—N1—C18	121.16 (18)	C11—C12—H12	120.6
C17—N1—C21	124.8 (2)	F1—C13—C12	119.0 (2)
C18—N1—C21	113.55 (18)	F1—C13—C14	118.4 (2)
O1—C1—C6	125.9 (2)	C12—C13—C14	122.6 (2)
O1—C1—C2	114.8 (2)	C13—C14—C15	118.4 (2)
C6—C1—C2	119.3 (2)	C13—C14—H14	120.8
O2—C2—C3	124.9 (2)	C15—C14—H14	120.8
O2—C2—C1	115.4 (2)	C14—C15—C10	121.3 (2)
C3—C2—C1	119.7 (2)	C14—C15—H15	119.4
C2—C3—C4	121.6 (2)	C10—C15—H15	119.4
C2—C3—H3	119.2	C9—C16—C17	126.5 (2)
C4—C3—H3	119.2	C9—C16—H16	116.8
C5—C4—C3	118.3 (2)	C17—C16—H16	116.8
C5—C4—C9	122.5 (2)	O3—C17—N1	121.9 (2)
C3—C4—C9	119.3 (2)	O3—C17—C16	121.1 (2)
C4—C5—C6	120.7 (2)	N1—C17—C16	117.03 (19)
C4—C5—H5	119.6	N1—C18—C19	110.90 (19)
C6—C5—H5	119.6	N1—C18—H18A	109.5
C1—C6—C5	120.4 (2)	C19—C18—H18A	109.5
C1—C6—H6	119.8	N1—C18—H18B	109.5
C5—C6—H6	119.8	C19—C18—H18B	109.5
O1—C7—H7A	109.5	H18A—C18—H18B	108.0
O1—C7—H7B	109.5	O4—C19—C18	111.38 (19)
H7A—C7—H7B	109.5	O4—C19—H19A	109.4
O1—C7—H7C	109.5	C18—C19—H19A	109.4
H7A—C7—H7C	109.5	O4—C19—H19B	109.4
H7B—C7—H7C	109.5	C18—C19—H19B	109.4
O2—C8—H8A	109.5	H19A—C19—H19B	108.0
O2—C8—H8B	109.5	O4—C20—C21	111.52 (19)
H8A—C8—H8B	109.5	O4—C20—H20A	109.3
O2—C8—H8C	109.5	C21—C20—H20A	109.3
H8A—C8—H8C	109.5	O4—C20—H20B	109.3
H8B—C8—H8C	109.5	C21—C20—H20B	109.3
C16—C9—C10	118.8 (2)	H20A—C20—H20B	108.0
C16—C9—C4	122.2 (2)	N1—C21—C20	109.63 (19)
C10—C9—C4	118.8 (2)	N1—C21—H21A	109.7
C15—C10—C11	117.8 (2)	C20—C21—H21A	109.7
C15—C10—C9	120.2 (2)	N1—C21—H21B	109.7
C11—C10—C9	121.8 (2)	C20—C21—H21B	109.7
C12—C11—C10	121.1 (2)	H21A—C21—H21B	108.2
C12—C11—H11	119.5		
			
C7—O1—C1—C6	9.9 (3)	C9—C10—C11—C12	173.5 (2)
C7—O1—C1—C2	−169.79 (19)	C10—C11—C12—C13	−0.5 (4)
C8—O2—C2—C3	4.3 (3)	C11—C12—C13—F1	−179.9 (2)
C8—O2—C2—C1	−176.16 (19)	C11—C12—C13—C14	1.2 (4)
O1—C1—C2—O2	1.1 (3)	F1—C13—C14—C15	−179.1 (2)
C6—C1—C2—O2	−178.6 (2)	C12—C13—C14—C15	−0.2 (4)
O1—C1—C2—C3	−179.38 (19)	C13—C14—C15—C10	−1.5 (4)
C6—C1—C2—C3	0.9 (3)	C11—C10—C15—C14	2.1 (3)
O2—C2—C3—C4	179.0 (2)	C9—C10—C15—C14	−172.5 (2)
C1—C2—C3—C4	−0.5 (3)	C10—C9—C16—C17	176.1 (2)
C2—C3—C4—C5	−0.7 (3)	C4—C9—C16—C17	−9.4 (4)
C2—C3—C4—C9	−179.5 (2)	C18—N1—C17—O3	−9.7 (3)
C3—C4—C5—C6	1.5 (3)	C21—N1—C17—O3	179.2 (2)
C9—C4—C5—C6	−179.8 (2)	C18—N1—C17—C16	168.3 (2)
O1—C1—C6—C5	−179.8 (2)	C21—N1—C17—C16	−2.8 (3)
C2—C1—C6—C5	−0.2 (3)	C9—C16—C17—O3	−54.5 (3)
C4—C5—C6—C1	−1.0 (3)	C9—C16—C17—N1	127.5 (2)
C5—C4—C9—C16	138.5 (2)	C17—N1—C18—C19	137.0 (2)
C3—C4—C9—C16	−42.8 (3)	C21—N1—C18—C19	−51.0 (3)
C5—C4—C9—C10	−47.0 (3)	C20—O4—C19—C18	−59.3 (3)
C3—C4—C9—C10	131.7 (2)	N1—C18—C19—O4	54.2 (3)
C16—C9—C10—C15	137.8 (2)	C19—O4—C20—C21	60.3 (2)
C4—C9—C10—C15	−36.9 (3)	C17—N1—C21—C20	−137.1 (2)
C16—C9—C10—C11	−36.6 (3)	C18—N1—C21—C20	51.2 (3)
C4—C9—C10—C11	148.7 (2)	O4—C20—C21—N1	−55.5 (3)
C15—C10—C11—C12	−1.1 (4)		

### Hydrogen-bond geometry (Å, °)

**Table:**

*D*—H···*A*	*D*—H	H···*A*	*D*···*A*	*D*—H···*A*
C19—H19A···O2^i^	0.99	2.57	3.279 (3)	129.
C11—H11···O2^ii^	0.95	2.58	3.478 (3)	158.
C15—H15···O3^iii^	0.95	2.38	3.216 (3)	147.
C21—H21A···O3^iv^	0.99	2.42	3.105 (3)	125.
C8—H8B···O4^v^	0.98	2.55	2.983 (3)	106.

Symmetry codes: (i) −*x*+1, −*y*+1, −*z*+1; (ii) −*x*+1, *y*+1/2, −*z*+1/2; (iii) −*x*+1, *y*−1/2, −*z*+1/2; (iv) *x*+1, *y*, *z*; (v) −*x*+2, −*y*+1, −*z*+1.
